# Evolving concepts in adjuvant/neoadjuvant therapy for resectable pancreas cancer

**DOI:** 10.1172/JCI191944

**Published:** 2025-07-15

**Authors:** John M. Bryant, Luis Ruffolo, Kevin Soares, Sarah Hoffe, Andrew M. Lowy

**Affiliations:** 1 Department of Radiation Oncology, Section of Gastrointestinal Radiation Oncology, Moffitt Cancer Center, Tampa, Florida, USA.; 2 Department of Surgery, Hepatopancreatobiliary Service, Memorial Sloan Kettering Cancer Center, New York, New York, USA.; 3 Department of Surgery, Division of Surgical Oncology, Moores Cancer Center, University of California San Diego, La Jolla, California, USA.

## Abstract

Despite advances in multidisciplinary oncology care, curing patients diagnosed with pancreatic duct adenocarcinoma (PDAC) remains all too uncommon. In this Review, we discuss evolving concepts to guide the care of patients with operable PDAC, focusing on adjuvant and neoadjuvant systemic therapies, the ever-controversial topic of radiation therapy, and the emerging role of cancer vaccines. Given the promise of biomarkers to better predict therapeutic response, the development of KRAS inhibitors, our ability to deliver higher doses of radiation therapy more precisely and safely, and the technology to rapidly produce highly personalized cancer vaccines, there is reason to expect that the guidelines for the care of our patients with operable PDAC will change rapidly in the next few years.

## Introduction

Despite many advances in the care of patients with pancreatic duct adenocarcinoma (PDAC), surgical resection remains vital for potential cure. Surgery can now be offered to more patients as a result of better systemic therapy, improved surgical techniques, and enhanced perioperative care. However, long-term survival after resection remains suboptimal. This Review will address unresolved questions about neoadjuvant and adjuvant chemotherapy, future cancer vaccines, and the controversial role of radiation therapy. Here, we aim to both answer and raise questions that combine biological concepts with current and forthcoming treatments as we look toward a future in which treatments will rely on a deeper understanding of the disease’s molecular biology, integrating its natural history and predicted therapy response.

## Neoadjuvant versus adjuvant therapy for resectable PDAC

Given the high rate of distant relapse after surgical resection of PDAC, systemic therapy is essential for curative treatment. Phase III studies have shown the benefit of adjuvant chemotherapy in prolonging disease-free survival (DFS) and overall survival (OS) over surgery alone, with regimens including FOLFIRINOX (folinic acid, fluorouracil, irinotecan, and oxaliplatin), gemcitabine/capecitabine, and gemcitabine/nab-paclitaxel (G-NP) improving OS over gemcitabine alone (1–5). An important consideration is that patients enrolled in adjuvant therapy trials represent a highly selected group, having successfully recovered from surgery. Neoadjuvant chemotherapy has been driven by several ideas: (a) Treating occult metastatic disease early may improve outcomes. (b) Pancreatic surgery is morbid, and postoperative complications may delay or prevent adjuvant therapy. Delivering therapy before surgery ensures chemotherapy exposure. (c) Chemotherapy prior to surgery is better tolerated. (d) Neoadjuvant therapy allows for response assessment. (e) Patients with refractory disease may avoid non-therapeutic surgery. (f) Preoperative therapy provides observation time to manage comorbidities and optimize patient fitness for successful surgery recovery.

Remarkably, even though neoadjuvant therapy has been delivered for decades, there remains extremely limited level 1 data directly comparing neoadjuvant to adjuvant therapy. Most studies of “neoadjuvant therapy” have used perioperative chemotherapy rather than total neoadjuvant chemotherapy. Table 1 summarizes landmark studies utilizing perioperative chemotherapy for PDAC (6). Several conclusions can be drawn. Patients treated with perioperative chemotherapy on average receive a greater percentage of intended chemotherapy in the preoperative period rather than the adjuvant setting. Most studies incorporating neoadjuvant therapy also demonstrated a reduction in positive lymph nodes and found an improvement in rates of margin-negative (R0) resection, both prognostic factors for OS.

Neoadjuvant therapy has many hypothesized advantages, but it poses multiple clinical challenges. These include the need for preoperative tissue diagnosis, preoperative biliary drainage in jaundiced patients, and venous access for chemotherapy. Monitoring patients during chemotherapy requires a coordinated multidisciplinary team to swiftly address treatment-related toxicity and biliary stent complications. Failure to address these issues may threaten patients’ candidacy for surgical resection. The NORPACT-1 phase II trial reported superior survival in patients undergoing pancreatectomy and adjuvant chemotherapy compared with intended 4 cycles of neoadjuvant FOLFIRINOX and adjuvant therapy (10). The study has faced numerous critiques, further emphasizing the need for level 1 data in this area. Two identically designed phase III trials comparing perioperative FOLFIRINOX (four cycles of neoadjuvant and two cycles of adjuvant therapy) are nearing full accrual in the United States (Alliance A021806) and Europe (PREOPANC-4) (Table 1). These studies will offer new insights into the value of these approaches for managing patients with resectable PDAC. Despite the results, given PDAC’s known inter- and intrapatient heterogeneity, many questions will remain — questions tracing back to our limited understanding of how systemic therapies impact PDAC biology. Major unanswered questions include:

*Can we identify biomarkers to select patients who should receive neoadjuvant therapy versus undergoing upfront surgical resection?* Often clinicians will select neoadjuvant therapy in those they judge to be at high risk for rapid relapse; however, specification of the prognostic factors predictive of rapid relapse remains imprecise. Molecular diagnostics such as circulating tumor DNA (ctDNA) are under active investigation to more precisely define radiographically occult disease. Early studies clearly suggest that detection of ctDNA prior to surgery and after neoadjuvant therapy are negative prognostic factors for resection. For example, data from the PANACHE01-PRODIGE48 trial demonstrated a median OS of 19.4 months in patients with a preoperative carbohydrate antigen 19-9 (CA19-9) level greater than 80 U/mL who also had detectable ctDNA and 30.2 months in the CA19-9–high or ctDNA^+^ group, and OS was not reached in the CA19-9–low or ctDNA^–^ group (log-rank *P* = 0.0069) (11). Similarly, in a study of resected PDAC patients, the median relapse-free survival was 13 months for patients in whom postoperative ctDNA was positive versus 22 months for those with negative ctDNA (*P* = 0.003) (12). Such data suggest that we may be able to define parameters predictive of very poor outcomes from surgery; such patients would be ideal candidates for neoadjuvant therapy using novel therapeutic approaches, among them vaccine strategies discussed in the next section. However, low sensitivity and thus a poor negative predictive value severely limit the utility of ctDNA at present (13).

*What is the optimal duration of neoadjuvant/adjuvant therapy?* Typically, preoperative therapy duration is guided by patient tolerance and clinical measures of response, including radiographic response and CA19-9 when detectable. No prospective studies have addressed this question specifically, while retrospective studies suggest that longer durations of neoadjuvant therapy are associated with better outcomes. However, these findings are biased, as treatment-related toxicity or ineffectiveness usually causes therapy to stop. Our inability to accurately define clinical benefit from neoadjuvant therapy remains a large gap that is currently being addressed by molecular diagnostics and imaging strategies. The most straightforward approach uses PET scans to assess for clinical response. A Mayo Clinic group reported that PET responses were prognostic for improved outcomes after neoadjuvant therapy (14). Other approaches under investigation include radiomics and molecular diagnostics like ctDNA (15). Improving our ability to understand the clinical benefit of neoadjuvant therapy in real time will help us individualize treatment duration more precisely, improving survival and reducing treatment-related toxicity.

Recently, SWOG 1505 noted greater median dose density of both modified FOLFIRINOX (mFOLFIRINOX) and G-NP when received preoperatively versus postoperatively (mFOLFIRINOX preoperative 87.5% vs. postoperative 59.6%, *P* < 0.001; G-NP preoperative 77.3% vs. postoperative 51.7%, *P* < 0.001) (7). In this study, dose density received was associated with median survival (8). Similarly, a recent retrospective single-institution study of 225 patients who underwent pancreatectomy for stage I/II PDAC found that regardless of treatment sequence, completion of at least 67% of the recommended number of chemotherapy cycles was associated with improved OS (median OS, 34.5) compared with less than 67% of cycles (median OS, 17.9 months; HR, 0.39; 95% CI, 0.24–0.64) (9). However, neoadjuvant therapy was associated with a greater likelihood of receiving more than 67% of prescribed cycles of chemotherapy. An analysis of ESPAC-3 data demonstrated that completion of all six cycles of planned adjuvant chemotherapy rather than early initiation was an independent prognostic factor for survival after resection (16). A recent retrospective study examined the duration of adjuvant chemotherapy after preoperative FOLFIRINOX and found that adjuvant treatment improved survival (17). The effect was most pronounced in patients receiving less than 4 months of preoperative therapy, consistent with prior data suggesting that receipt of a minimum of two-thirds of prescribed therapy is beneficial.

*Is it beneficial to switch neoadjuvant chemotherapy?* For patients who receive perioperative therapy, a common clinical dilemma involves determining criteria for changing therapy either during the neoadjuvant component or in the adjuvant setting. There are prospective data to guide such decisions, but a recent meta-analysis of five retrospective studies involving 863 patients who underwent neoadjuvant therapy for localized PDAC found that 20% of patients underwent chemotherapy switching (18). Of these, 42% underwent curative-intent resection, and their survival was comparable to that of patients receiving first-line chemotherapy. Three phase II trials (NCT03322995, NCT04594772, and NCT04539808, ClinicalTrials.gov) are currently in progress in the United States evaluating chemotherapy switching for patients with potentially resectable PDAC. All are non-randomized phase II studies using FOLFIRINOX, each with slightly different criteria for switching to G-NP. While these studies will provide additional new data, ultimately only a randomized trial can definitively address the question of whether changing therapy in the neoadjuvant setting is of benefit, and unfortunately these studies’ relevance may be overrun by the emergence and integration of predictive biomarkers and more effective systemic therapies, namely KRAS inhibitors, as discussed in the next section.

*How will our evolving understanding of PDAC biology and KRAS inhibitors change our approach to adjuvant/neoadjuvant therapy?* Identifying basal and classical transcriptional subtypes of PDAC has led to studies on whether this biology predicts response or resistance to systemic therapies (19). Early data suggested that the basal subtype may be more resistant to FOLFIRINOX versus G-NP, with ongoing studies of biomarker-selected neoadjuvant therapy, including one using the Purity Independent Subtyping of Tumors (PurIST) classifier to differentiate basal versus classical subtypes (NCT0468331) (20). Recent preclinical data suggest that PDAC has distinct tumor-intrinsic kinomes related to basal and classical subtyping, which have implications for therapeutic response (21). For instance, basal-subtype tumors were more reliant on EGFR signaling and thus more responsive to EGFR inhibitors. Emerging data suggest that classical-subtype tumors may be more resistant to KRAS inhibition, a hypothesis that needs clinical testing (22). With pan-KRAS and KRASG12D-specific inhibitors entering late-phase trials for advanced disease, the next frontier will be their incorporation into the treatment of resectable disease. Given that response rates in chemorefractory advanced PDAC have ranged from 20% to 30% and disease control rates have approached 90%, it is hypothesized that KRAS inhibitors will markedly improve outcomes for resectable disease (23, 24). We need to understand whether there is a biological rationale for timing these therapies relative to surgical resection. Effective therapy prior to an operation could improve margin-negative surgery and reduce procedure-related tumor cell dissemination. Alternatively, reducing tumor cell burden via surgery may enhance the effectiveness of adjuvant KRAS inhibitors. Addressing such questions with preclinical studies is challenging, necessitating next-generation clinical trials that integrate these biomarkers to understand the place of these emerging therapies in managing patients with resectable PDAC, and that integrate them with cytotoxic regimens with proven, though modest, benefit.

## Radiation therapy for resectable PDAC

Despite improvements in surgical technique and more common use of neoadjuvant chemotherapy, the rate of positive surgical margins and locoregional recurrence after PDAC resection remains high. Integration of radiation therapy aims to improve local disease control and enhance margin-negative resection rates. While evidence suggests it can achieve these objectives, defining its precise role in PDAC care remains challenging. We will discuss recent data on curative-intent radiation therapy, future technologies, and biological settings, clarifying its place in patient management.

For resectable PDAC, a surgery-first approach carries a 30%–50% incidence of positive surgical margins (2). Concurrent chemoradiation therapy (CRT) with standard fractionation (1.8–2 Gy per day; biologically effective dose, α/β = 10 [BED10] = 59.47 Gy) has long been the standard postoperative approach (25, 26). Prospective data integrating treatment schemata with BED10 less than 70 Gy have historically shown mixed results regarding survival benefits with adjuvant radiotherapy (27, 28). ESPAC-1 failed to show OS benefit, and LAP-07 similarly found no OS improvement in locally advanced settings (5, 29). RTOG 9704 randomized 451 patients to receive adjuvant CRT (50.4 Gy in 28 fractions) with 5-fluorouracil or gemcitabine and found no survival difference between arms (30). Secondary analyses showed improved survival of patients adhering to the radiotherapy protocol (20.9 vs. 17.5 months) and better outcomes in node-negative disease and postoperative CA19-9 levels less than 180 U/mL (31, 32). This led to RTOG 0848, which showed improved DFS for the entire cohort but no significant OS improvement (27 vs. 31 months, HR 0.96, 90% CI 0.79–1.18, *P* = 0.38) (33). In node-negative patients, however, CRT demonstrated notable benefits with 5-year OS rates of 48% versus 29% (HR for interaction 2.34, 90% CI 1.27–4.29, *P* = 0.0063) and 5-year DFS rates of 47% versus 19% (HR for interaction 2.05, 90% CI 1.16–3.61, *P* = 0.014). While these findings suggest a possible benefit of CRT in node-negative disease, the overall role of adjuvant radiation therapy in improving survival remains uncertain.

Whether median OS is the ideal endpoint to evaluate radiation therapy’s value is debated. In a disease with high systemic relapse, expecting local therapy to markedly improve survival may be unrealistic. RTOG 0848 data are consistent with this: patients at lower systemic relapse risk (node negative) did have improved survival after adjuvant CRT, while those at higher risk (node positive) did not. With more effective systemic therapies, enhancing local therapy with radiation will remain a relevant question.

## Radiation therapy for borderline or locally advanced PDAC

For patients with borderline or locally advanced unresectable PDAC, achieving resection is crucial for survival. Only 15%–20% of locally advanced pancreatic cancer (LAPC) patients and 50%–60% of borderline resectable pancreatic cancer (BRPC) patients undergo resection, but they achieve outcomes comparable to those of initially resectable patients if R0 resection is achieved (34–42). The PREOPANC-1 trial provided randomized evidence for neoadjuvant radiotherapy in resectable and borderline resectable PDAC. This phase III trial randomized 246 patients to receive either gemcitabine-based CRT (36 Gy in 15 fractions) followed by surgery and adjuvant gemcitabine, or upfront surgery with adjuvant gemcitabine. Results showed a slight improvement in median survival (15.7 vs. 14.3 months, *P* = 0.025) and a higher rate of R0 resections (72% vs. 43%, *P* < 0.001) (43). However, subgroup analysis showed no survival benefit for resectable PDAC, and the use of an outdated chemotherapy regimen limits broader applicability. As a result, many centers consider both upfront surgery followed by chemotherapy and neoadjuvant radiotherapy viable for resectable PDAC (44).

Neoadjuvant radiotherapy is typically delivered using conventional fractionation over 5 to 6 weeks (45–51). Standard fractionation with BED10 less than 70 Gy has not shown significant survival benefits for unresectable PDAC, prompting investigation of hypofractionated radiotherapy (HFRT) and stereotactic body radiotherapy (SBRT) as alternatives to deliver higher BED10 regimens up to 100 Gy (52–56). The interval from standard fractionation radiotherapy to surgery allows more time for disease progression. A 2-week accelerated fractionation schedule, incorporating an intraoperative boost, was explored to shorten treatment time (57, 58). This accelerated method reduced overall treatment duration and applied HFRT in PDAC early.

Modern image-guided radiotherapy techniques have allowed safe dose escalation while reducing treatment sessions. HFRT relies on daily imaging to ensure proper setup. SBRT, delivered in five or fewer fractions, is well suited for PDAC regimens. Shorter treatment durations help reduce delays to surgery or systemic therapy, and stereotactic techniques enable safer delivery of higher doses. Despite improvements, the role of these modern approaches in PDAC treatment remains debated, highlighting the need for innovation and well-designed clinical trials (59–63).

Stereotactic magnetic resonance–guided adaptive radiation therapy (SMART) integrates MRI guidance with adaptive planning, offering real-time tumor tracking and daily plan adjustments. SMART delivers ablative doses while minimizing toxicity to surrounding structures (64). Clinical data on SMART for BRPC and LAPC are encouraging. In a study by Rudra et al., ablative SMART improved local control and OS compared with non-adaptive SBRT in unresectable disease (65). A single-center study reported a 96% R0 resection rate following SMART, with no grade 2+ acute toxicities and excellent postoperative outcomes (66). Median progression-free survival exceeded 13 months, underscoring SMART’s potential to enhance surgical and oncologic outcomes.

Two recent studies exploring SMART in upfront unresectable PDAC after induction chemotherapy showed promising efficacy and safety. A Danish phase II trial with 28 LAPC patients reported a 21% resection rate and a median OS of 20.8 months, which was improved by 7.7 months in those resected (37). The phase II multicenter SMART trial (NCT03621644) enrolled 136 upfront unresectable PDAC patients, showing a 22.8-month median OS and a 94% one-year OS rate (67). SMART was well tolerated in both trials, aligning with retrospective reports (65, 69–71).

Ablative dose radiation continues to garner support for continued exploration in prospective randomized trials after neoadjuvant chemotherapy for upfront unresectable PDAC.

Active clinical trials exploring novel approaches incorporating radiation therapy and surgery in PDAC are highlighted in Table 2. The PANDAS–PRODIGE 44 trial (NCT02676349) evaluates BRPC patients randomized to receive either neoadjuvant modified FOLFIRINOX alone or with conventional CRT at 50.4 Gy with capecitabine.

Tailoring patient selection for radiotherapy may enhance outcomes while minimizing toxicity. Mutations in DNA damage response (DDR) pathways are linked to improved outcomes with platinum-based chemotherapies and PARP inhibitors in PDAC, though their impact on radiotherapy response is less well understood (72). Early data suggest increased radiosensitivity in DDR tumors in preclinical models and retrospective studies (73, 74). Future studies targeting patients with DDR mutations will be essential to evaluate whether incorporating radiotherapy into multimodal treatments can enhance outcomes.

The transcription factor NRF2, often upregulated in PDAC as a result of KRAS mutations, contributes to chemotherapy and radiotherapy resistance by activating antioxidant DNA response elements and reducing reactive oxygen species, critical mediators of radiation-induced DNA damage (75). Upregulated NRF2 expression is associated with poorer survival rates in patients receiving radiation therapy (76). Strategies inhibiting NRF2 or its pathways, like glutamine metabolism, have shown potential in preclinical models to enhance sensitivity to radiation and chemotherapy (75, 76). These findings highlight opportunities to address resistance mechanisms in PDAC, expanding the pool of resectable patients after neoadjuvant therapy and improving overall outcomes in this challenging disease.

In summary, trials incorporating non-ablative doses of radiotherapy have failed to show significant impact and have caused controversy regarding the benefit of radiotherapy in the treatment of PDAC. Recent innovations in the precision of radiation oncology delivery techniques have led to safe dose escalation, suggesting that local-control improvements will only be identified if the tumor dose achieved is in the range of 72–100 Gy. Future work is needed to clarify which strategies can overcome biological tumor resistance and determine how best to coordinate systemic therapy integration with ablative dose regimens for patients with upfront unresectable PDAC.

## Cancer vaccines as adjuvant PDAC therapy

Beyond conventional cytotoxic neoadjuvant and adjuvant therapies, recent advances in cancer vaccines for PDAC patients undergoing curative resection have shown promise. PDAC’s immune desert or immune-excluded microenvironment poses a barrier to immune-based therapies (77–80). Cancer vaccines offer a strategy to overcome these barriers by priming the immune system to target tumor-associated and tumor-specific antigens (TAAs and TSAs). Deploying this approach in patients with minimal residual disease may confer the advantage of circumventing microenvironmental barriers to an adaptive immune response in micrometastases, like mature tumor stroma or recruited suppressive immune populations (81).

## Key concepts in vaccine therapy for PDAC

The application of vaccines as anticancer therapies dates to 1893, when William Coley, a surgeon in New York’s Memorial Hospital, observed tumor regression in patients injected with bacterial toxins (82). Advances in molecular biology and recombinant DNA technology have enabled the identification of TAAs and TSAs, transforming cancer vaccines into a personalizable therapeutic modality. By the late 20th century, investigators began exploring peptide-based, whole-cell, and dendritic cell (DC) vaccines, aiming to activate the immune system against tumor-specific targets (83). In PDAC, interest in vaccine therapy intensified with the identification of shared tumor antigens like KRAS, MUC1, and WT1 (84–86). Unfortunately, phase III studies in vaccine therapies for unresectable PDAC have failed to translate immune responses into improved clinical outcomes (87–89).

PDAC employs redundant mechanisms to escape immune detection and elimination, including downregulating antigen presentation and suppressing T cell activity through the recruitment of Tregs, myeloid-derived suppressor cells, and tumor-associated macrophages (78). This process often goes hand in hand with immune editing, a dynamic mechanism in which immunogenic tumor clones are selectively eliminated, leaving behind resistant variants (90). The low rate of tumor mutations in PDAC reduces the chances that an adaptive immune response will be induced owing to poor antigenicity (91).

**Figure 1:**
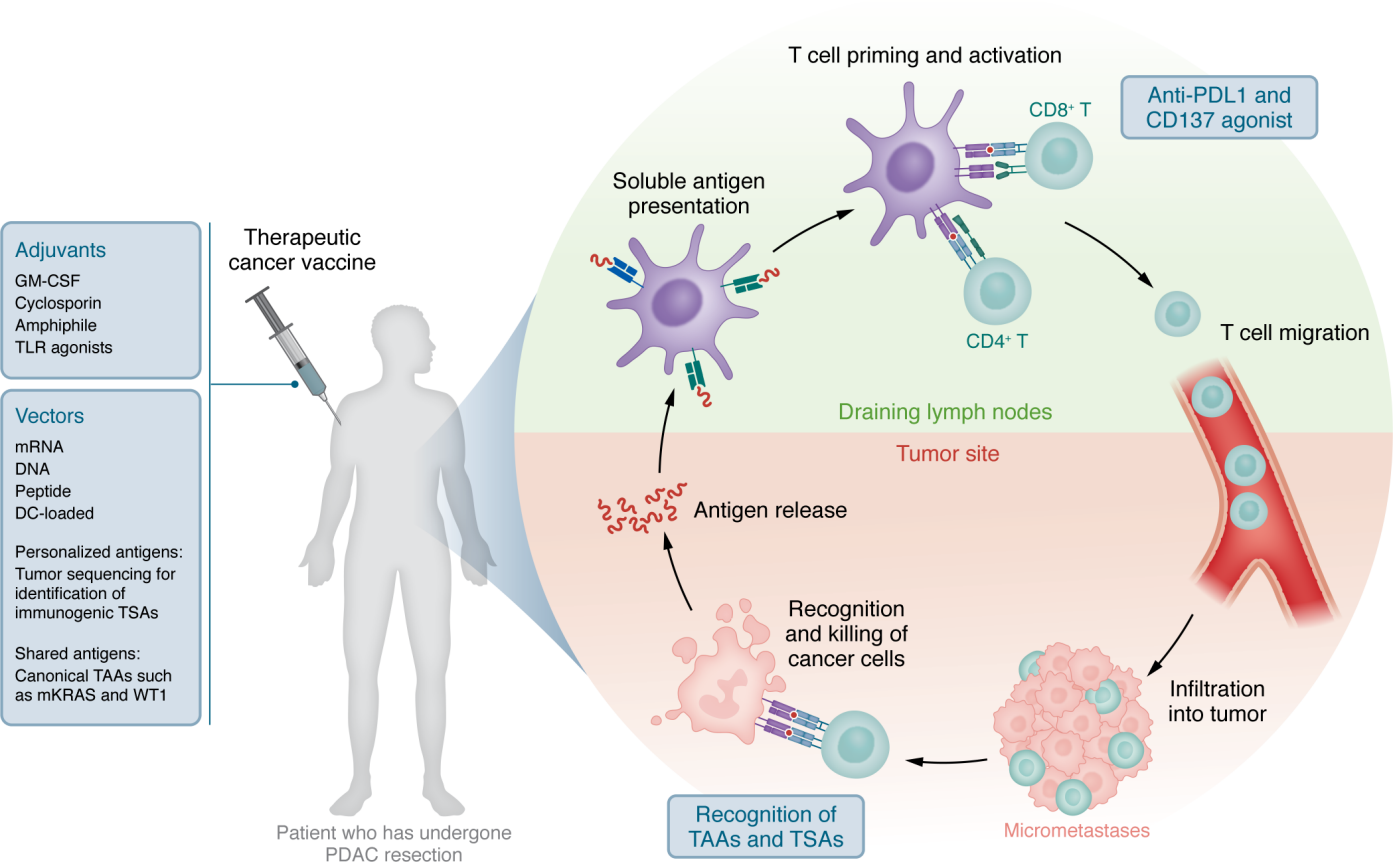
Mechanism of vaccine-induced cancer-specific immune response. The adjuvant or minimal residual disease setting represents an attractive approach for vaccine therapy in PDAC. Advantages include avoiding significantly higher tumor burden and its associated immunosuppression in the advanced/metastatic setting as well as optimizing the ratio of effector T cells to tumor cells.

Priming antigen-specific immune responses is critical to overcome the immunosuppressive nature of PDAC. DNA, RNA, and peptide vaccines are designed to present TAAs and TSAs to the host immune system, enabling the activation of CD4^+^ helper T cells and CD8^+^ cytotoxic T cells. These vaccines encode antigenic material translated or processed within the host, leading to antigen presentation through MHC class I and II pathways. This cross-presentation mechanism is essential for robust CD8^+^ T cell activation while supporting CD4^+^ T cell priming, which is critical for sustained immune responses and immune memory (Figure 1). DC-based vaccines involve the ex vivo differentiation and activation of autologous monocytes into highly effective antigen-presenting cells. These DCs are loaded with target peptides or tumor antigens in vitro, enabling them to prime and activate T cells upon infusion into the patient.

Adjuvants play a pivotal role in enhancing antigenicity of vaccine strategies. Toll-like receptor (TLR) agonists, such as CpG oligonucleotides, mimic pathogen-associated molecular patterns to stimulate innate immunity and enhance T cell priming, and low-dose cyclophosphamide has been adapted to deplete Tregs. Thus, the integration of adjuvants into cancer vaccine strategies can be critical to achieve a robust and sustained antitumor immune response (92–95). A review of the literature offers perspective into the measurable immunogenicity of reported adjuvant vaccine platforms, permitting an assessment of therapeutic potential (Table 3).

Shared-antigen vaccines focus on targeting antigens overexpressed across cancers, including PDAC. For example, mutated KRAS is found in over 90% of PDAC cases and is a prime target for peptide-based vaccines like ELI-002 (96). Similarly, MUC1, an aberrantly glycosylated glycoprotein, and WT1, a transcription factor overexpressed in PDAC, have been incorporated into vaccine platforms such as GVAX and DC-based therapies. In contrast, neoantigen vaccines leverage the unique mutation-derived antigens of individual tumors. Platforms like autogene cevumeran use mRNA technology to deliver these personalized neoantigens, eliciting potent and tumor-specific T cell responses (97). The selection of neoantigens involves identifying mutation-derived epitopes with high affinity for a patient’s MHC molecules. Advances in next-generation sequencing and bioinformatics have greatly facilitated this process, enabling the rapid identification of immunogenic targets from tumor samples (97, 98).

## Pancreas cancer vaccine therapy for patients with minimal residual disease

Shared-antigen vaccine platforms have been at the forefront of immunotherapy development in PDAC with minimal residual disease (MRD), targeting widely expressed TAAs (Table 3). One such effort was the phase II randomized trial of GI-4000 (NCT00300950), a yeast-based vaccine targeting mutant KRAS (mKRAS). In this study, 176 patients received either the vaccine or placebo alongside adjuvant gemcitabine after surgical resection. While the trial did not demonstrate significant differences in recurrence-free survival (RFS) or OS between treatment groups, the immune responses varied by resection status. Among patients with R1 resections, 40% of those receiving the vaccine exhibited immune responses compared with only 8.3% in the placebo arm (*P* = 0.062). Conversely, immune activation was limited in the R0 resection cohort, underscoring the challenge of stimulating robust immunity in patients with MRD (99).

Another vaccine, TG01 (NCT02261714), demonstrated significant immunogenicity in a phase I/II trial targeting mKRAS. This peptide-based vaccine was administered with GM-CSF and gemcitabine to 32 patients with resected stage I/II PDAC. Ninety-four percent of participants mounted a positive immune response, with delayed-type hypersensitivity testing or T cell proliferation assays confirming T cell activation. Median OS for the cohort was 33.3 months, and patients completing five or six cycles of gemcitabine had a median OS of 37.0 months. Although early allergic reactions led to dose modifications, subsequent participants tolerated the vaccine well, reinforcing its potential for integration into broader treatment regimens (100).

ELI-002 2P, evaluated in the AMPLIFY-201 trial (NCT04853017), introduced a novel lymph node–targeted amphiphile peptide vaccine designed against G12D and G12R KRAS mutations. This study enrolled 25 patients (20 with PDAC and 5 with colorectal cancer) with MRD confirmed by ctDNA or elevated CA19-9 or CEA (a general tumor marker). Remarkably, 84% of patients exhibited immune responses. Vaccine responders (defined as vaccine-specific T cell response above the median fold increase over baseline) showed significant tumor marker reductions compared with non-responders. Additionally, median RFS in responders was not reached, compared with 4.01 months in the non-responders (HR, 0.14; *P* = 0.0167). In summary, clinical efficacy correlated with T cell response, supporting the rationale for further studies deploying shared-antigen vaccines in the MRD setting (101).

The Cy-CVAX platform study at Johns Hopkins Medical Center (NCT02451982) investigated the efficacy of GVAX in combination with immune checkpoint blockade and a costimulatory antibody against 4-1BB (CD137) in resected PDAC (102). The trial used a neoadjuvant and adjuvant design with three arms: (a) GVAX and low-dose cyclophosphamide (Cy-GVAX), (b) Cy-GVAX with nivolumab (anti–PD-1 antibody), and (c) Cy-GVAX combined with nivolumab and urelumab (anti-CD137 agonist). Immune monitoring demonstrated significant intratumoral immune activation, with arm C achieving the highest CD8^+^CD137^+^ T cell infiltration (70%) compared with arms A and B (*P* = 0.003). Clinical outcomes revealed encouraging DFS rates, with median DFS of 13.9 months in arm A, 15.0 months in arm B, and 33.5 months in arm C. Similarly, OS appeared to improve in arm C, though sample size limitations precluded definitive statistical comparisons. Perhaps most notably, the trial highlights the impact of serial analysis of immune responses to vaccine-adjuvant combinations, facilitating the sequential addition of synergistic immunotherapies to overcome mechanisms of treatment resistance (102).

Personalized neoantigen vaccines, which leverage unique mutation-derived antigens, have also demonstrated promise in PDAC. In one such trial investigating the mRNA-based vaccine autogene cevumeran (NCT04161755), patients who underwent resection received eight priming doses of adjuvant autogene cevumeran combined with a single dose of atezolizumab followed by adjuvant FOLFIRINOX (103). Sixteen of 20 patients received personalized vaccines. Immune monitoring revealed neoantigen-specific responses in 50% of vaccinated patients, characterized by polyfunctional T cell activation. As in the AMPLIFY-201 trial, vaccine responders exhibited prolonged RFS compared with non-responders (median RFS not reached vs. 13.4 months; HR, 0.14; 95% CI, 0.03–0.6; *P* = 0.007) (104). Notably, one patient developed a new elevation in CA19-9 while receiving adjuvant chemotherapy after vaccine priming. Cross-sectional imaging demonstrated a new liver nodule that prompted suspicion of metastasis; however, biopsy of this lesion showed a dense lymphoid infiltrate that included all 15 of the patient’s known vaccine-expanded CD8^+^ T cell populations. Digital droplet PCR analysis of the liver lesion demonstrated rare cells with mutated *TP53* alleles synonymous with the patient’s primary tumor, yet no viable carcinoma on histology. This lesion and CA19-9 elevation ultimately resolved, suggesting the potential for vaccine-induced T cells to eradicate micrometastases (103).

Another study at Washington University in St. Louis explored neoantigen-based peptide and DNA vaccines (NCT03122106) (105). Neoantigens were identified through whole-exome sequencing and delivered via synthetic peptides or plasmid DNA encoding prioritized neoepitopes. Among participants, 100% of DNA vaccine recipients and nearly all peptide vaccine recipients mounted neoantigen-specific T cell responses. Long-term outcomes remain to be reported pending final analysis of these cohorts (105).

In the PCNAT-01 trial (NCT03558945), a peptide-based vaccine combined with the adjuvant poly-ICLC was evaluated against patient-specific neoantigens (106). The preliminary abstracts reported RFS rates of 60%, 52.5%, and 43.8% at 3, 4, and 5 years, respectively. Immune profiling demonstrated the expansion of cytotoxic T cell clonotypes and functional gene enrichment in responders. These data further underscore the immunogenic potential of personalized neoantigen platforms, while awaiting confirmation of therapeutic benefit (106).

DC-based vaccines represent another avenue for immunotherapy in PDAC. The REACtiVe DC vaccine trial (EudraCT 2018-003222-92), conducted in Rotterdam, evaluated autologous DCs pulsed with tumor lysates in 38 patients who had undergone surgery and chemotherapy (107). Immune monitoring demonstrated that 100% of patients exhibited delayed-type hypersensitivity reactions to the vaccine peptides and vaccine-driven activation of CD4^+^ central memory, effector memory, and effector memory RA^+^ T cells, as well as CD4^+^ and CD8^+^ T cell activation, upon exposure to the vaccine peptide ex vivo. At 2 years, RFS was 64%, while OS reached 83%, meeting the prespecified phase II primary endpoint (107).

## Challenges and future directions in adjuvant PDAC vaccines

Building on the promising early-phase vaccine studies in MRD, several phase II trials aim to further validate these approaches. The AMPLIFY-7P trial (NCT05726864) is investigating the next-generation amphiphile vaccine targeting multiple KRAS mutations, building on findings from the phase I AMPLIFY-201 study, and focusing on robust T cell responses as a correlate for relapse-free survival. The autogene cevumeran vaccine platform demonstrated potent neoantigen-specific T cell responses and encouraging RFS in its phase I study (NCT04161755) and has entered a phase II trial (NCT05968326) for a large cohort of patients with resectable head of pancreas cancer.

The adjuvant/MRD space is an attractive setting for testing vaccines in PDAC. It offers an improved ratio of effector T cells to target tumor cells and allows vaccine therapy before the establishment of immunosuppressive mechanisms, such as loss of MHC class I expression in the epithelial compartment and recruitment of tumor-supportive stroma. Nonetheless, these trials face considerable scientific hurdles. Shared-antigen vaccines demonstrate significant immunogenicity, but their clinical efficacy may be limited by central immune tolerance mechanisms in the MRD setting. Conversely, personalized neoantigen vaccines and DC-based platforms offer tailored, potent immune responses but require logistically complex and resource-intensive personalization.

Vaccine platforms have intrinsic pros and cons to consider in the designing of therapeutic trials. DNA-based vaccines are more stable than mRNA-based platforms but raise concerns about integration into the host DNA. DNA vaccines often need strong adjuvants because of impaired immunogenicity compared with mRNA-backboned approaches (108). Tissue-informed versus off-the-shelf target antigens trade ease of manufacturing for personalized optimal antigen-MHC cross-presentation.

Still, integrating synergistic adjuvants and checkpoint inhibitors holds promise for enhancing these therapies’ effectiveness, leading to sustained immune responses. Future treatment algorithms may query a patient’s tumor mutations and consider vaccines with off-the-shelf targets such as mKRAS in addition to non-shared neoantigens, weighing the therapeutic benefits of each approach for individual patients, considering their MHC alleles and probabilities of response. Sequential studies on mechanisms of treatment failure, like the platform trial at Johns Hopkins, provide a blueprint for incremental efficacy. As we accumulate safety experience with vaccine strategies, applying these therapies into window-of-opportunity trials can help parse the immune dynamics that follow vaccination with robust tissue analysis on resected tissue, permitting sequential combinations of immunotherapies. Importantly, vaccine-based pancreas cancer clinical trials have yet to definitively improve OS, but studies are ongoing. These trials will ultimately inform strategies to circumvent redundant and unrecognized pathways of immune evasion in the post-vaccination tumor microenvironment. Further investments in window-of-opportunity, adjuvant, and MRD clinical trials are essential to leverage these findings and optimize vaccine strategies for PDAC in adjuvant and neoadjuvant treatment paradigms.

## Conclusions

For years, the primary questions regarding management of resectable PDAC have revolved around the potential benefits of neoadjuvant versus adjuvant therapy and whether there is a role for radiotherapy. Despite these unanswered queries, advances in PDAC science, drug development, and technology are set to soon reshape patient care. The incorporation of prognostic and predictive biomarkers will soon inform decisions on sequencing and the choice of agents. As we better understand individual cancer risk for local versus distant recurrence and potential sensitivity to radiation therapy, we can rationally tailor patient treatments. The promising data from KRAS inhibitor and vaccine trials suggest that these will soon join our therapeutic armamentarium. The future landscape will prioritize individual tumor biology over anatomy in treatment plans for resectable PDAC.

## Figures and Tables

**Table 3:**
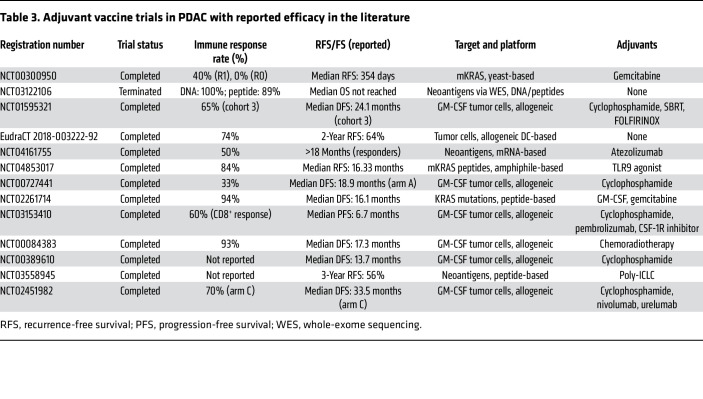
Adjuvant vaccine trials in PDAC with reported efficacy in the literature

**Table 2:**
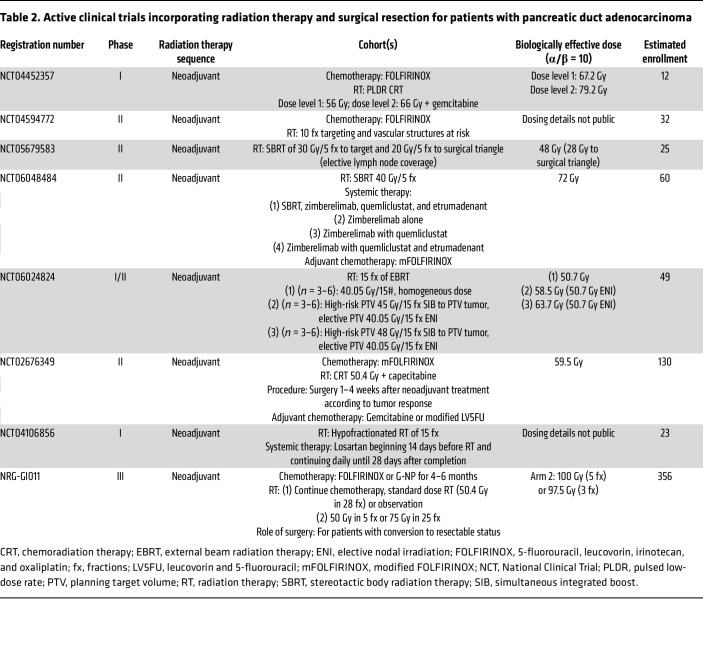
Active clinical trials incorporating radiation therapy and surgical resection for patients with pancreatic duct adenocarcinoma

**Table 1:**
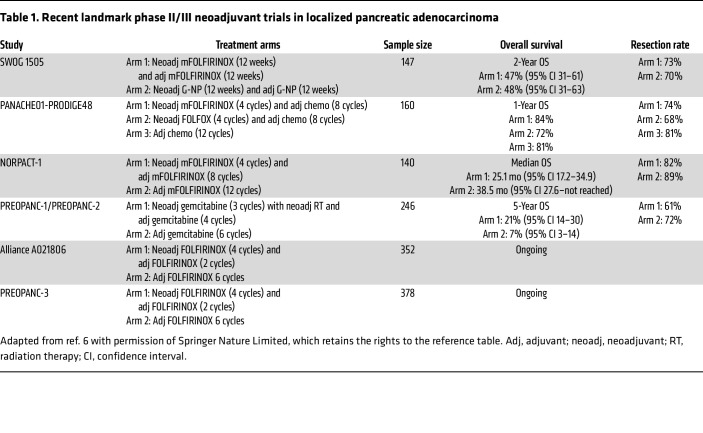
Recent landmark phase II/III neoadjuvant trials in localized pancreatic adenocarcinoma

## Version 1 07/15/2025

Electronic publication
